# Apple Cider Vinegar Powder Mitigates Liver Injury in High-Fat-Diet Mice via Gut Microbiota and Metabolome Remodeling

**DOI:** 10.3390/nu17132157

**Published:** 2025-06-28

**Authors:** Qiying Ding, Dai Xue, Yilin Ren, Yuzheng Xue, Jinsong Shi, Zhenghong Xu, Yan Geng

**Affiliations:** 1 Key Laboratory of Carbohydrate Chemistry and Biotechnology, Ministry of Education, School of Life Science and Health Engineering, Jiangnan University, Wuxi 214122, China; 6221504002@stu.jiangnan.edu.cn (Q.D.);; 2 Affiliated Children’s Hospital of Jiangnan University, Wuxi 214000, China; 3 Department of Gastroenterology, Affiliated Hospital of Jiangnan University, Wuxi 214125, China; 4 College of Biomass Science and Engineering, Sichuan University, Chengdu 610065, China

**Keywords:** high-fat diet, chronic liver injury, apple cider vinegar, gut microbiota, cecal metabolites, gut–liver axis, lipid metabolism

## Abstract

**Background/Objectives**: High-fat-diet (HFD) consumption drives chronic liver injury via gut dysbiosis and metabolic disturban. Apple cider vinegar, rich in polyphenols and organic acids, shows potential in metabolic regulation. This study aimed to investigate whether apple cider vinegar powder (ACVP) alleviates HFD-induced liver injury by modulating the gut–liver axis. **Methods**: For 12 weeks, C57BL/6 J mice received daily ACVP gavage while being fed a HFD. A series of biological assessments were conducted, including systemic metabolic evaluations (body weight, serum alanine aminotransferase (ALT)/aspartate aminotransferase (AST), and lipid/glucose levels), hepatic steatosis (hematoxylin and eosin (H&E) staining), intestinal microbiome characterization (16S rRNA gene genomic analysis), and comprehensive metabolite profiling of cecal contents (non-targeted metabolomics). Pearson correlation networks integrated multi-omics data. **Results**: ACVP attenuated HFD-induced weight gain by 26.3%, hepatomegaly and dyslipidemia, as well as reduced hepatic lipid vacuoles and serum ALT (48%)/AST (21.5%). ACVP restored gut microbiota diversity, enriching *Muribaculaceae*. Cecal metabolomics identified 38 HFD-perturbed metabolites reversed by ACVP, including indolelactate, hyocholate, and taurocholic acid. the Kyoto encyclopedia of genes and genomes (KEGG) analysis revealed ACVP-mediated recovery of linoleic acid metabolism. Correlation networks linked *Akkermansia* to anti-inflammatory metabolites (e.g., trans-ferulic), while Desulfobacterota correlated with pro-inflammatory oxylipins (e.g., 12,13-dihydroxy-9Z-octadecenoic acid (DHOME)). **Conclusions**: ACVP mitigates HFD-induced liver injury by remodeling gut microbiota, restoring microbial metabolites, and enhancing gut–liver crosstalk.

## 1. Introduction

High-fat-diet (HFD) consumption has been widely recognized as a key contributor to metabolic disorders and obesity, a pressing global health crisis associated with an increased risk of cardiometabolic diseases, including type 2 diabetes, cardiovascular disorders, and non-alcoholic fatty liver disease [1]. In young generations, including children and adolescents, obesity has become astonishingly widespread. This trend forecasts long-lasting problems for various body functions and imposes a weighty socio-economic burden [2]. Chronic liver injury induced by a HFD, characterized by hepatic steatosis, oxidative stress, and low-grade inflammation, exemplifies the intricate interplay between dietary patterns, gut dysbiosis, and systemic metabolic dysfunction. Epidemiological studies indicate that HFD-induced liver damage is not only associated with lipid accumulation, but also with gut dysbiosis and altered intestinal metabolite profiles, which exacerbate systemic metabolic dysfunction through the gut–liver axis [3,4,5,6]. For instance, a HFD disrupts intestinal barrier integrity by increasing reactive oxygen species (ROS) production from gut microbiota, leading to bacterial translocation and hepatic inflammation [7]. Additionally, HFD reshapes microbial communities, reducing beneficial taxa (e.g., *Bacteroidetes* and *Lactobacillus*) while promoting pathogenic bacteria, such as *Enterobacteriaceae*, which further aggravates liver injury by modulating bile acid metabolism and short-chain fatty acid (SCFA) synthesis [8,9]. Notably, the emerging evidence highlights the bidirectional relationship between obesity and gut microbiota composition, where microbial dysbiosis both drives and perpetuates metabolic disturbances, creating a vicious cycle that complicates therapeutic interventions [10].

The emerging evidence suggests that dietary interventions targeting gut microbiota and their metabolites hold promise for mitigating HFD-related liver damage. Probiotics and prebiotics, for example, have shown efficacy in restoring microbial balance and improving lipid metabolism via adenosine 5′-monophosphate (AMP)-activated protein kinase (AMPK) signaling pathways and the suppression of pro-inflammatory cytokines [3]. Fermented, organic, acid-rich foods, like vinegar, show potential to modulate gut microbiota and strengthen intestinal barrier integrity [11,12]. Apple cider vinegar (ACV), a natural product containing acetic acid and polyphenols, has demonstrated anti-obesity and hepatoprotective effects in preliminary studies, possibly through its prebiotic properties and the regulation of microbial-derived metabolites, like SCFAs [13,14,15]. However, the specific mechanisms by which ACV ameliorates HFD-induced chronic liver injury remain underexplored, particularly its role in reshaping the gut microbiota and associated metabolic pathways.

In this study, we aim to investigate whether dietary apple cider vinegar powder (ACVP) supplementation can alleviate chronic liver injury in mice fed a HFD. We examined the gut microbiota structure and gut metabolite profiles of these mice to see if changes correlated with liver injury amelioration. We hypothesize that ACVP intervention attenuates hepatic damage by restoring microbial diversity, regulating intestinal metabolism, and suppressing pathogenic bacterial translocation. Our findings may provide novel insights into dietary strategies for preventing HFD-related metabolic complications through gut–liver axis modulation.

## 2. Materials and Methods

### 2.1. Materials

The ACVP was produced by Lvjie Co., Ltd. (Yantai, Shandong, China), which contains 0.3 mg of total polyphenols and 48 mg of acetic acid per g. The AIN-93G diet and 60% high-fat diet were both purchased from Jiangsu Xietong Pharmaceutical Bio-Engineering Co., Ltd.

### 2.2. Ethical Consideration

The Institutional Animal Care and Use Committee at Jiangnan University authorized the animal study protocol (Approved No. JN. No. 20230530c1100915[240]).

### 2.3. Animal Treatments

**Figure 1:**
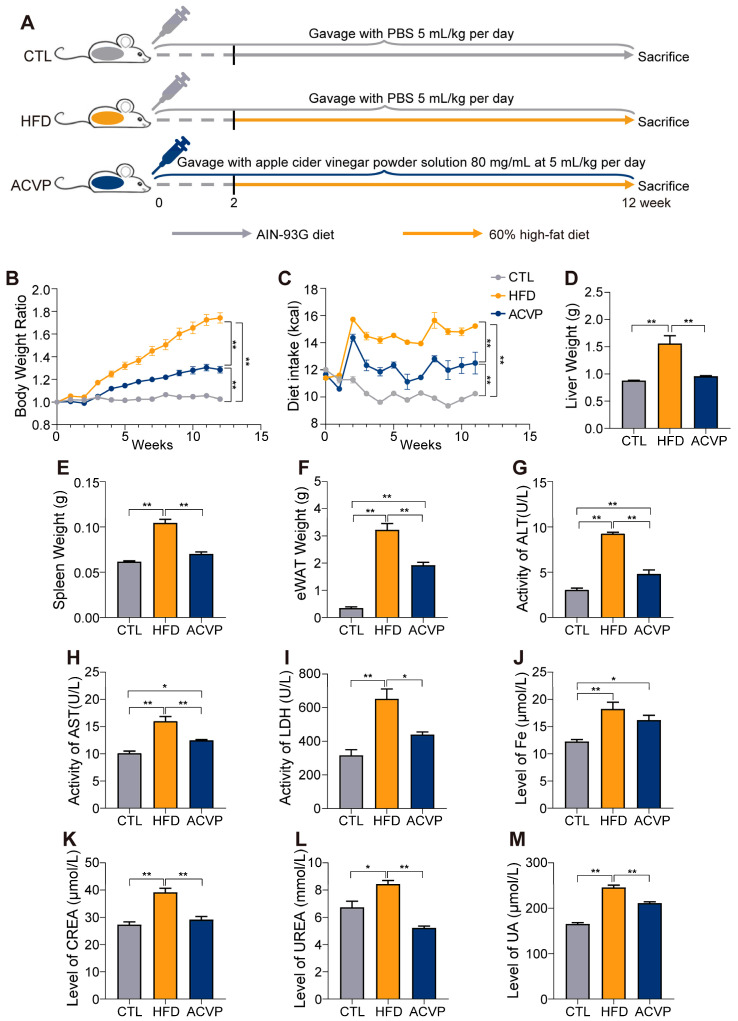
Apple cider vinegar powder (ACVP) supplementation affects the physiological status induced by high-fat diets (HFDs). (**A**) Schematic diagram of mouse groups, n = 6. (**B**) Body weight ratio and (**C**) dietary intake of the experiment cycle. (**D**) Liver weight, (**E**) spleen weight, and (**F**) epididymal white adipose tissue (eWAT) weight of the mice at the time of dissection. (**G**) Serum alanine aminotransferase (ALT), aspartate aminotransferase (AST) (**H**), lactate dehydrogenase (LDH) (**I**), Fe (**J**), creatinine (CREA) (**K**), urea (UREA), (**L**) and uric acid (UA) (**M**) levels in mice. Error bars represent mean ± SEM, * *p* < 0.05, and ** *p* < 0.01.

The experimental design details are graphically illustrated in Figure 1A. We procured 18 male C57BL/6 J mice, aged 9 weeks, from GemPharmatech Co., Ltd. (Nanjing, China). Every animal fulfilled the inclusion criteria of this experiment. These mice were maintained under specific pathogen-free conditions with a regulated 12 h light/dark cycle, humidity levels at 40 ± 5%, and temperature maintained in the range of 20–22 °C. The animals had free access to both water and food. After the acclimatization period, the mice were arbitrarily distributed into three distinct groups (n = 6): one group on a standard AIN-93G diet (CTL), one on a 60% high-fat diet (HFD), and one group on the HFD supplemented with the ACVP solution [16,17]. During the first two weeks, all groups of animals were fed with AIN-93G diets and received corresponding gavage interventions (PBS for the CTL and HFD groups, and apple cider vinegar powder for the ACVP group) to adjust their health in advance. Starting from week 2, mice in the HFD and ACVP groups were fed with a 60% high-fat diet, while those in the CTL group remained on the AIN-93G diet. Throughout the experiment, all mice were administered a PBS or ACVP solution via intragastric gavage daily. In animal experiments, each group was assigned 6 mice to ensure adequate statistical power and account for potential variability among individuals, while also considering the practical feasibility and ethical guidelines regarding animal usage. The sample size was determined based on a post hoc power analysis (false discovery rate (FDR) < 0.1). The primary measure was differential metabolite levels. Utilizing the online platform MetaboAnalyst, the analysis determined a requisite sample size of 6 for each group to achieve an 80% power level between the HFD and CTL groups, and a 70% power level between the ACVP and HFD groups (Figure S1) [18]. The composition of diets was provided in Table 1. A HFD is a common method to induce chronic liver injury in animal models. It can simulate the dietary habits of people with chronic liver diseases to some extent, and the mechanism of liver injury induced by a high-fat diet is relatively clear, which facilitates the study of the therapeutic effects of ACVP. The administered volume of the ACVP solution was 80 mg/mL at 5 mL per kg of body weight. Converting this to a human equivalent dosage, using standard body surface area calculations [19], equates to a daily intake of 2g of ACVP for a 60 kg adult. ACVP is a concentrated form of ACV, which is more convenient for storage and use. Moreover, its composition is more stable, which can ensure the consistency of the experimental results. At the end of the 12-week study period, the mice were sacrificed to collect samples of serum, liver, and colon tissues for analysis. The experimenters and Institutional Animal Care and Use Committee at Jiangnan University were aware of the animal group allocation throughout the experimental period.

### 2.4. Histological Evaluation

Liver tissues were fixed, paraffin-embedded, and hematoxylin and eosin (H&E)-stained per standard protocols. Histological analysis combined digital scanning (3DHISTECH Pannoramic MIDI, Hungary) with a light microscopy (Olympus BX53, Japan) evaluation.

### 2.5. Biochemical Analysis of Serum Samples

Venous blood specimens underwent centrifugation (3500× *g*, 15 min) for serum separation, followed by cryopreservation at −80 °C pending biochemical evaluation. Hepatic functional markers (aspartate aminotransferase (AST), alanine aminotransferase (ALT), and lactate dehydrogenase (LDH)) were determined using standardized enzymatic assays (Nanjing Jiancheng Bioengineering Institute, China), while extended metabolic profiling encompassing iron homeostasis (Fe), renal functional indices (creatinine (CREA), urea (UREA), and uric acid (UA)), and lipid–glucose metabolism parameters (triglycerides (TG), total cholesterol (CHOL), high-density lipoprotein cholesterol (HDL-C), low-density lipoprotein cholesterol (LDL-C), and glucose (GLU)) was performed on the EXC 400 automated analyzer (Zybio Inc., China) with dedicated reagent systems, requiring 5 μL of serum per analytical run.

### 2.6. Total RNA Extraction and Real-Time Quantitative PCR

Total RNA was isolated from samples using a Trizol reagent (Thermo Fisher Scientific, USA), followed by cDNA synthesis with a reverse transcription kit (Takara, Japan) according to the manufacturer’s instructions. Quantitative PCR was executed on a MyIQ2 real-time PCR system (Bio-Rad, USA) with iTaq Universal SYBR Green Supermix (Bio-Rad, USA). The relative gene expression was calculated using the 2^−ΔΔCt^ method, with *β-actin* as the endogenous control for normalization. Data are presented as fold-changes relative to the control group (CTL). Primer sequences are provided in Supplementary Table S1.

### 2.7. Sequencing and Subsequent Analysis of the 16S rRNA Gene

After collecting fecal samples for 12 weeks, DNA was extracted using the DNeasy PowerSoil Pro Kit (Qiagen, USA). The V3–V4 hypervariable regions of bacterial 16S rRNA were amplified through PCR and sequenced on an Illumina NovaSeq 6000 platform (paired-end, 2 × 250 bp). Amplified products (4–5 pM) were pooled for paired-end sequencing. Raw sequencing data were processed through DADA2 to generate amplicon sequence variants (ASVs) by resolving single-nucleotide differences. Taxonomic annotation was performed against the SILVA 138.1 database using QIIME2 (v2019.4) with a 95% similarity threshold, implemented via a pre-trained Naïve Bayes classifier.

Beta diversity analysis employed principal coordinate analysis (PCoA) based on unweighted UniFrac distances. Microbial–metabolite correlations were calculated using Pearson coefficient in R (v3.6.3), with relative abundance matrices generated from normalized ASV counts. Full methodological details, including primer sequences and bioinformatics parameters, are provided in the Supplementary Materials.

### 2.8. Non-Targeted Metabolic Analysis of Cecal Contents

Untargeted metabolomic analysis was performed using UHPLC-MS/MS with a Q Exactive Focus mass spectrometer (Thermo Fisher Scientific, USA) and an ACQUITY UPLC^®^ HSS T3 column (150 mm × 2.1 mm, 1.8 μm) (Waters, Milford, MA, USA). The experimental procedures and analysis methods were adapted from a previous study [11]. Compare CTL vs. HFD, ACVP vs. HFD, and ACVP vs. CTL groups pairwise. Use Student’s *t*-test (*p*-value < 0.05) and VIP ≥ 1 to filter differential metabolites.

### 2.9. Statistical Analysis

Data are expressed as mean ± SEM and analyzed via GraphPad Prism version 8.0 (CA, USA) and the online platform MetaboAnalyst. One-way ANOVA (with Dunnett’s post hoc test) compared the three groups. Unpaired two-tailed Student’s *t*-test compared two groups. *p* < 0.05 was significant.

## 3. Results

### 3.1. ACVP Attenuates Pathological Changes and Metabolic Dysregulation in HFD-Fed Mice

Chronic HFD feeding for 12 weeks induced significant weight gain in mice compared to the CTL group. Weekly monitoring revealed that HFD-fed mice exhibited a progressive increase in body weight, whereas ACVP supplementation partially reversed this trend, with the ACVP group showing intermediate values between CTL and HFD throughout the intervention period (Figure 1A,B, Table S2). At the study endpoint, the final body weight of the HFD group was significantly higher than both the CTL and ACVP groups (Figure 1B). The food intake was monitored throughout the experiment and converted into calories. The calorie intake significantly increased in the HFD group, while ACVP intervention reduced the calorie intake (Figure 1C). Consistent with systemic metabolic overload, HFD-fed mice displayed marked hepatomegaly, splenomegaly, and epididymal white adipose tissue (eWAT) hypertrophy. ACVP significantly reversed these results (Figure 1D–F).

HFD-induced hepatic dysfunction was confirmed by elevated serum markers of liver injury, including ALT, AST, and LDH. ACVP intervention significantly reduced these markers, indicating the partial restoration of hepatic homeostasis (Figure 1G–I). HFD feeding disrupted systemic iron metabolism, as evidenced by elevated serum Fe levels in the HFD group compared to CTL (Figure 1J). Additionally, HFD-induced renal stress was reflected in increased serum CREA, UREA, and UA. ACVP supplementation ameliorated these perturbations, suggesting systemic metabolic benefits beyond hepatic protection (Figure 1K–M). These findings demonstrate AVCP dietary intervention can improve pathological remodeling and metabolic disorders.

### 3.2. ACVP Mitigates HEPATIC Steatosis, Restores Lipid–Glucose Homeostasis, and Attenuates Inflammatory Signaling in HFD-Fed Mice

**Figure 2:**
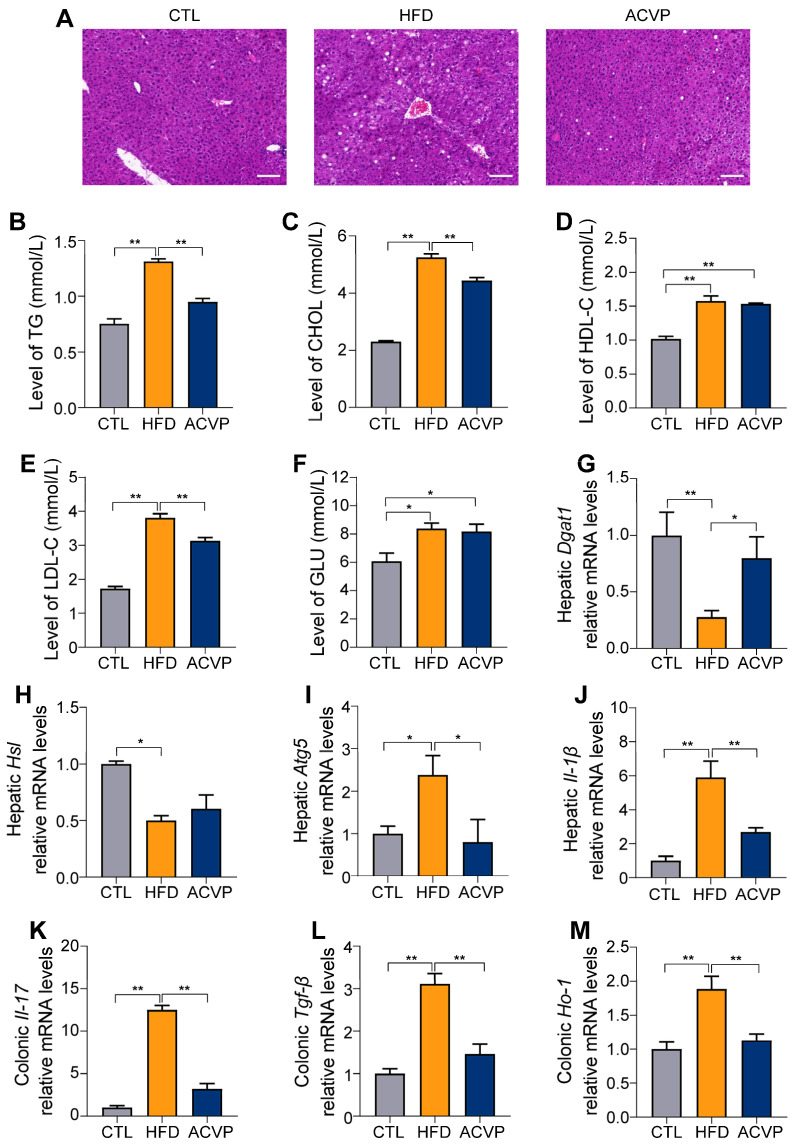
Apple cider vinegar powder (ACVP) supplementation prevents liver injury and intestinal barrier damage induced by high-fat diets (HFDs). (**A**) The representative sections of liver stained with hematoxylin and eosin (scale bars, 100 μm). (**B**) Serum triglycerides (TGs), total cholesterol (CHOL) (**C**), high-density lipoprotein cholesterol (HDL-C) (**D**), low-density lipoprotein cholesterol (LDL-C), (**E**) and glucose (GLU) (**F**) levels in mice. (**G**–**J**) Relative gene expression of *Dgat1*, *Hsl*, *Atg5*, and *Il-1β* in the liver and (**K**–**M**) *Il-17*, *Tgf-β*, and *Ho-1* in the colon. Error bars represent mean ± SEM, * *p* < 0.05, and ** *p* < 0.01.

Histological analysis of liver tissue via H&E staining revealed severe microvesicular steatosis in HFD-fed mice, characterized by abundant lipid vacuoles, whereas the CTL group exhibited normal hepatic architecture. ACVP intervention markedly reduced lipid droplet accumulation, with only scattered vacuoles observed (Figure 2A). These morphological changes aligned with the serum metabolic profiles: HFD significantly elevated TG, CHOL, HDL-C, LDL-C, and GLU. ACVP supplementation reversed these alterations (Figure 2B–F). HFD-induced hepatic lipid accumulation was mechanistically linked to upregulated lipogenesis and impaired lipolysis. Quantitative PCR analysis demonstrated a significant decrease in hepatic *Dgat1* transcriptional expression in the HFD group, a key enzyme in triglyceride synthesis, which was attenuated by ACVP (Figure 2G). Moreover, HFD suppressed *Hsl* transcriptional expression, critical for lipolysis (Figure 2H). Additionally, HFD-fed mice exhibited elevated *Atg5* transcriptional expression, suggesting dysregulated autophagy, which was normalized by ACVP (Figure 2I).

Furthermore, HFD triggered pro-inflammatory responses in both liver and colon tissues, with elevated *Il-1β*, *Il-17*, and *Tgf-β* transcriptional expression compared to CTL. ACVP treatment suppressed these cytokines (Figure 2J–L). Concurrently, HFD-induced oxidative stress, reflected by upregulated hepatic *Ho-1*, was alleviated by ACVP (Figure 2M).

### 3.3. ACVP Modulates Gut Microbiota Composition and Restores Microbial Diversity in HFD-Fed Mice

**Figure 3:**
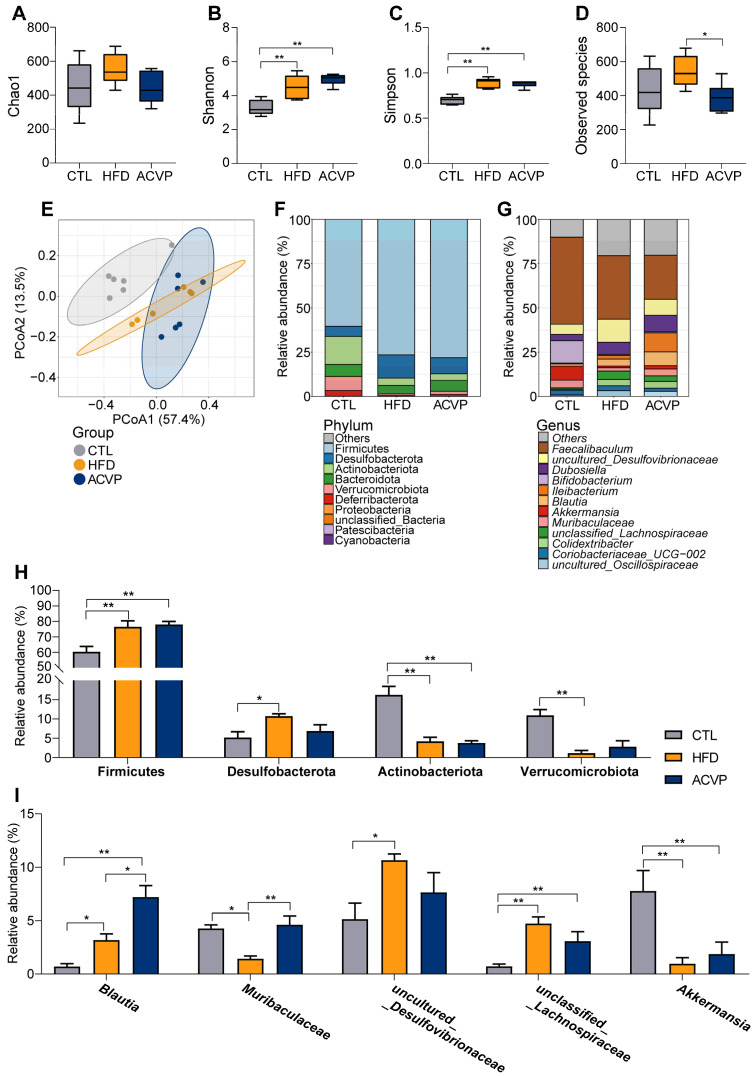
The effect of the intake of apple cider vinegar powder (ACVP) on the gut microbial structure in mice fed with high-fat diets (HFDs). α diversity index (**A**) Chao 1, (**B**) Shannon, (**C**) Simpson, and (**D**) observed species of intestinal microbiota in each group. (**E**) Principal coordinate analysis (PCoA) plot. (**F**) Bacterial taxonomic profiling of the gut microbiota at the phylum and genus (**G**) levels. (**H**) Relative abundances of Fimicutes, Desulfobacterota, Actinobacteriota, and Verrucomicrobiota. (**I**) Relative abundances of *Blautia*, *Muribaculaceae*, *uncultured_Desulfovibrionaceae*, *unclassified_Lachnospiraceae*, and *Akkermansia*. Error bars represent mean ± SEM, * *p* < 0.05, and ** *p* < 0.01.

Chronic HFD feeding significantly changed gut microbial diversity, as evidenced by increased alpha diversity indices in the HFD compared to CTL. Specifically, Shannon and Simpson indices were increased. ACVP intervention decreased observed species (Figure 3A–D). PCoA of β diversity showed clear differences in clustering between the three groups (Figure 3E). At the phylum level, HFD-fed mice exhibited a marked increase in Firmicutes and Desulfobacterota, alongside reduced Actinobacteriota and Verrucomicrobiota. ACVP supplementation reversed these trends, decreasing Desulfobacterota while elevating Verrucomicrobiota (Figure 3F,H). Genus-level analysis further highlighted microbial remodeling. HFD suppressed beneficial taxa, such as *Muribaculaceae* and *Akkermansia*, while promoting pathobionts, like *uncultured_Desulfovibrionaceae* and *unclassified_Lachnospiraceae*. ACVP intervention restored *Muribaculaceae* and *Akkermansia*, while reducing *uncultured_Desulfovibrionaceae and unclassified_Lachnospiraceae*. It should be noted that only the restoration of *Muribaculaceae* is significant. In addition, after receiving the HFD, the abundance of *Blautia* increases, which is more significant after ACVP intervention (Figure 3G,I).

*Muribaculaceae*, a promising beneficial bacterial family, can produce SCFAs from both endogenous sources (like mucin glycans) and exogenous polysaccharides (such as dietary fibres). It interacts mutually with well-known probiotics, like Bifidobacterium and Lactobacillus. Previous research has shown that higher levels of *Muribaculaceae* are linked to the beneficial effects of plant-based diets on conditions like inflammatory bowel disease, obesity, and type 2 diabetes [20]. The HFD-induced enrichment of sulfate-reducing Desulfobacterota and *Desulfovibrionaceae* correlates with elevated hydrogen sulfide production, a metabolite implicated in gut inflammation [21]. Previous studies demonstrated that Blautia-enriched microbiota correlate with reduced serum LDL-C and increased fecal bile acid excretion, aligning with our findings that ACVP may amplify lipid homeostasis [22,23].

### 3.4. ACVP Reverses HFD-Induced Dysbiosis by Modulating Key Gut Microbial Taxa Linked to Metabolic and Inflammatory Pathways

**Figure 4:**
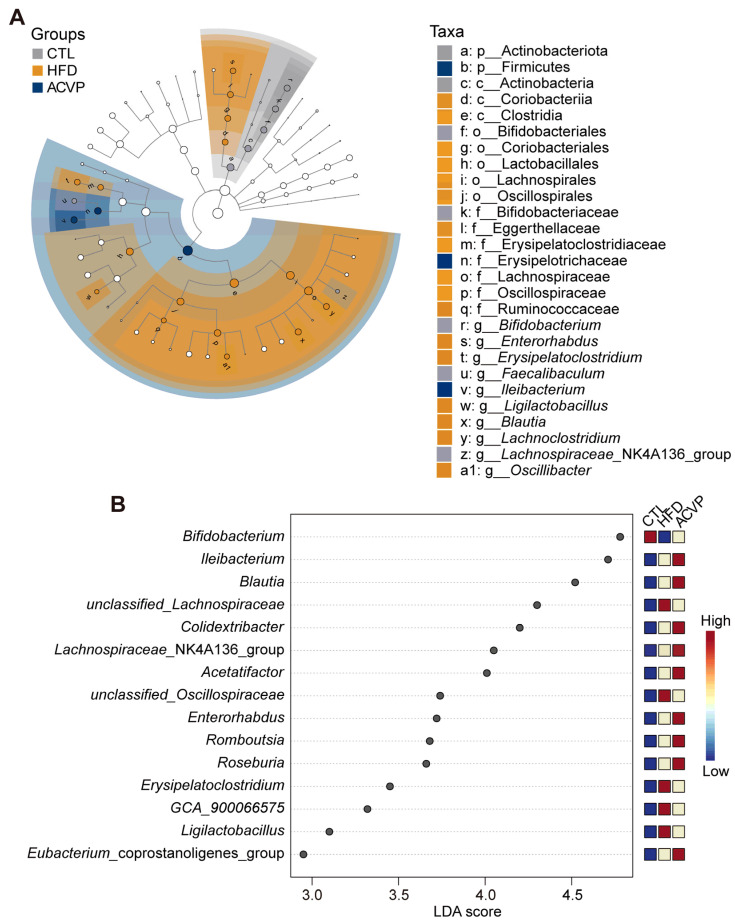
Effect of the administration of apple cider vinegar powder (ACVP) on dominant microorganisms in mice fed with high-fat diets (HFDs). Linear discriminant analysis (LDA) of effect size (LEfSe) analysis of gut microbiota, including (**A**) the cladogram and (**B**) distribution histogram based on the LDA score.

Linear discriminant analysis (LDA) of effect size (LEFSe) identified 15 genera with significant differences among the CTL, HFD, and ACVP groups (LDA score > 2.5). The LDA cladogram (Figure 4A) and score plot (Figure 4B) highlight *Bifidobacterium* as the most enriched genus in the CTL group, while *unclassified_Lachnospiraceae*, *unclassified_Oscillospiraceae*, *Erysipelatoclostridium*, *GCA-900066575*, and *Ligilactobacillus* are significantly increased in the HFD group. ACVP supplementation partially restored these taxa to intermediate levels between CTL and HFD. Notably, genera, such as *Roseburia*, *Eubacterium*_coprostanoligenes_group, and *Romboutsia*, showed a further elevation in ACVP compared to the HFD, suggesting the unique microbial remodeling effect of ACVP intervention.

### 3.5. ACVP Restores Cecal Metabolite Pathways Involving Lipid Metabolism, Bile Acid Transport, and Amino Acid Metabolism

**Figure 5:**
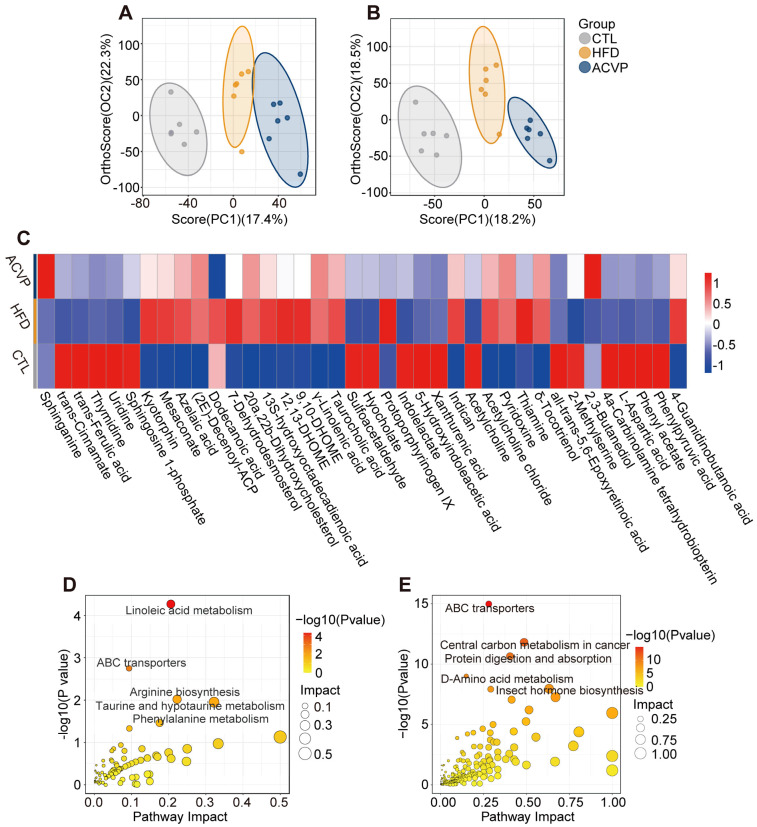
The effects of the intake of apple cider vinegar powder (ACVP) on metabolites of cecal contents in mice fed with high-fat diets (HFDs). Orthogonal partial least squares discriminant analysis (OPLS-DA) score plots of positive-ion (**A**) and negative-ion (**B**) modes in mouse cecum metabolites. (**C**) Heatmap analysis of different metabolites in cecal contents by ACVP. Kyoto encyclopedia of genes and genomes (KEGG) pathway enrichment analysis of differential metabolites in HFD vs. control (CTL) (**D**) and ACVP vs. HFD (**E**) mice.

Orthogonal partial least squares discriminant analysis (OPLS-DA) of cecal metabolites demonstrated a clear separation among CTL, HFD, and ACVP groups in both positive-ion (Figure 5A) and negative-ion modes (Figure 5B), indicating significant metabolic reprogramming induced by HFD ACVP. Among 106 significantly altered metabolites (Student’s *t*-test, *p*-value < 0.05, and VIP ≥ 1) (Table S3), 38 exhibited consistent trends. HFD feeding reduced levels of sphingolipid intermediates (sphinganine and sphingosine 1-phosphate), phenolic acids (trans-cinnamate and 5-hydroxyindoleacetic acid), while increasing pro-inflammatory lipids (12,13-dihydroxy-9Z-octadecenoic acid (DHOME), 9,10-DHOME) and bile acids (taurocholic acid) compared to CTL. ACVP intervention reversed these tendencies (Figure 5C). The Kyoto encyclopedia of genes and genomes (KEGG) pathway analysis of CTL vs. HFD mice highlighted a significant enrichment in linoleic acid metabolism, ABC transporters, and taurine/hypotaurine metabolism (Figure 5D). ACVP vs. HFD analysis revealed distinct pathway enrichment, including central carbon metabolism in cancer, protein digestion/absorption, and D-amino acid metabolism (Figure 5E). ACVP-enriched D-amino acid metabolism may modulate gut microbial peptidoglycan remodeling, enhancing immune tolerance [24].

### 3.6. ACVP Rebalances Gut–Liver Axis Crosstalk via Microbial–Metabolite Networks in HFD-Fed Mice

**Figure 6:**
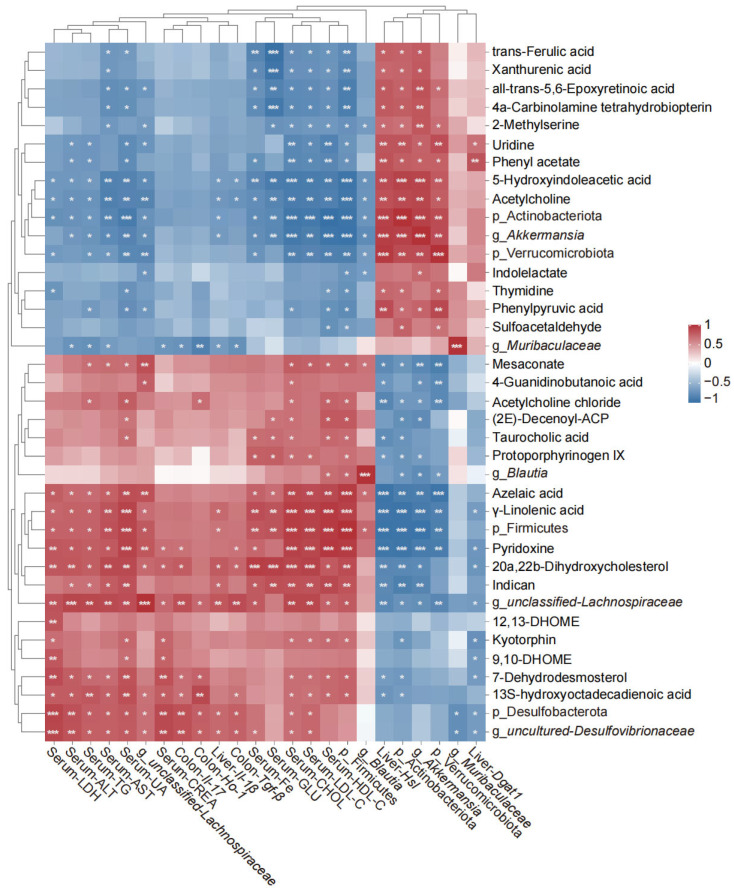
Pearson correlation analysis among the serum biomarkers, liver parameters, colon parameters, gut microbiota, and microbial metabolites in mice fed with three different treatments (control (CTL), high-fat diets (HFDs), and apple cider vinegar powder (ACVP)). Blue represents a negative correlation and red represents a positive correlation. * *p* < 0.05, ** *p* < 0.01, and *** *p* < 0.001.

Pearson correlation analysis unveiled robust associations among serum/liver injury markers, gut microbial taxa, and cecal metabolites, delineating a gut–liver axis regulatory network disrupted by a HFD and modulated by ACVP (Figure 6). Serum ALT, AST, LDH, and pro-inflammatory markers (*Il-17*, *Il-1β*, *Tgf-β*) exhibited strong positive correlations with Firmicutes, Desulfobacterota, and pro-inflammatory metabolites, such as taurocholic acid and 12,13-DHOME. Conversely, these markers negatively correlated with *Muribaculaceae*, *Akkermansia*, and anti-inflammatory metabolites, like trans-ferulic acid. Firmicutes and *unclassified_Lachnospiraceae* positively correlated with taurocholic acid and 13S-hydroxyoctadecadienoic acid, aligning with their roles in bile acid deconjugation and pro-inflammatory oxylipin production. These taxa also associated with serum TG, CHOL, LDL-C, and HDL-C, suggesting microbial-driven lipogenesis and hyperlipidemia. Protoporphyrinogen IX and indican, both markers of oxidative heme metabolism and uremic toxins, are linked to Desulfobacterota-mediated hydrogen sulfide overproduction. *Akkermansia* and Actinobacteriota strongly correlated with trans-ferulic acid, a polyphenol metabolite enhancing tight junction stability.

## 4. Discussion

The present study demonstrates that daily intervention with ACVP attenuates HFD-induced chronic liver injury through the multi-dimensional modulation of the gut–liver axis. Our findings reveal that ACVP not only ameliorates hepatic steatosis and systemic metabolic dysfunction, but also restores gut microbiota diversity and cecal metabolite profiles, establishing a novel link between dietary polyphenols, microbial remodeling, and host metabolic homeostasis. ACVP intervention significantly reduced hepatic lipid accumulation, as evidenced by the decreased liver weight, serum TG/CHOL levels, and hepatic *Dgat1* transcriptional expression. The reason may be that ACVP can reduce calories intake by suppressing appetite. This aligns with prior studies showing that acetic acid—a key component of ACV—enhances AMPK phosphorylation, thereby suppressing lipogenesis and promoting fatty acid oxidation [25,26]. Notably, ACVP downregulated *Dgat1* (a key enzyme in triglyceride synthesis) while upregulating *Hsl* (critical for lipolysis), suggesting a dual regulatory effect on lipid metabolism. Similar effects were observed in mice fed diets enriched with poorly absorbed long-chain SFAs, which improved hepatic lipid profiles through microbiota-dependent mechanisms [27]. Furthermore, the restoration of *Atg5* to baseline levels implies that ACVP normalizes autophagy flux, a process often dysregulated in HFD-induced metabolic stress [25].

HFD-induced gut dysbiosis, characterized by reduced *Muribaculaceae* and *Akkermansia* alongside the enrichment of *Desulfovibrionaceae*, was partially reversed by ACVP. Moreover, ACVP intervention can significantly increase the abundance of *Blautia*, a genus within the Lachnospiraceae family known for its role in lipid metabolism. *Muribaculaceae* can generate SCFAs from endogenous sources (mucin glycans) and exogenous polysaccharides (dietary fibers), and it has cross-feeding interactions with probiotics like *Bifidobacterium* and *Lactobacillus* [20]. The ACVP-mediated enrichment of *Akkermansia muciniphila*, a mucin-degrading bacterium associated with improved gut barrier integrity and anti-inflammatory effects, aligns with its established role in mitigating metabolic syndrome and type 2 diabetes. *Akkermansia* enhances mucus layer thickness, stimulates tight junction proteins, and modulates host immunity via Toll-like receptor signaling, thereby improving insulin sensitivity and reducing systemic inflammation [28,29]. Population-based cohort studies have demonstrated that *Blautia* is inversely associated with pro-atherogenic, small HDL particles and positively correlates with bile acid metabolism, suggesting its potential to improve lipid profiles by modulating cholesterol homeostasis and fatty acid oxidation [30]. The ACVP-driven restoration of these aligns with improved glucose homeostasis and reduced hepatic steatosis, suggesting crosstalk between microbial composition and host metabolism. These findings are consistent with reports that dietary polyphenols selectively promote beneficial taxa while inhibiting pro-inflammatory species and mitigating “leaky gut” syndrome [31,32,33]. The marked reduction of *Bifidobacterium* in HFD-fed mice aligns with prior studies linking its depletion to impaired gut barrier integrity and dysregulated lipid metabolism [34]. Conversely, ACVP-mediated suppression of *unclassified_Lachnospiraceae* and *Erysipelatoclostridium* (associated with TMA/TMAO generation) supports its anti-inflammatory role [34]. Elevated abundance of *Roseburia* (a butyrate producer) and *Eubacterium_coprostanoligenes_group* (critical for cholesterol metabolism) highlights their potential in lipid homeostasis. The unexpected increase in *Ligilactobacillus* may reflect ACVP’s prebiotic properties, as this genus degrades polyphenols into bioactive metabolites that attenuate oxidative stress [7].

Non-targeted metabolomics revealed that ACVP restored HFD-depleted metabolites, such as indolelactate, hyocholate, and taurocholic acid. At the hepatic level, antioxidant components of ACVP (such as polyphenols and acetic acid derivatives) mitigate oxidative stress by scavenging ROS and activating Nrf2-mediated antioxidant pathways, thereby reducing lipid peroxidation and DNA damage. This aligns with the studies demonstrating that antioxidants counteract drug- and chemical-induced hepatotoxicity by preserving mitochondrial function and suppressing pro-inflammatory cytokines [35]. Indolelactate, a microbial tryptophan metabolite, binds to the aryl hydrocarbon receptor (AhR) to suppress NF-κB-driven inflammation [27,36], while hyocholate restoration suggests improved FXR signaling for bile acid regulation [36,37]. Reduced sphinganine and sphingosine 1-phosphate in HFD-fed mice indicate impaired membrane integrity, consistent with gut barrier dysfunction [34]. ACVP also reversed HFD-driven increases in pro-inflammatory oxylipins (12,13-DHOME) [38], and oxidative stress markers (xanthurenic acid), likely through direct ROS scavenging by polyphenols [39]. Pearson correlation analysis highlighted robust associations between *Akkermansia*, anti-inflammatory metabolites (e.g., xanthurenic acid), and improved hepatic function. Conversely, *Desulfovibrionaceae* and taurocholic acid formed a pro-inflammatory cluster linked to elevated ALT and LDL-C.

## 5. Conclusions

Our study supports the potential of ACVP as a cost-effective dietary intervention for metabolic syndrome. The hepatoprotective effects of ACVP—through reducing hepatic lipid synthesis, enhancing β-oxidation, and suppressing oxidative stress—may indirectly lower cardiovascular risk by improving lipid profiles and attenuating endothelial dysfunction. These effects position ACVP as a potential adjunct therapy for dyslipidemia. The observed improvements in glucose tolerance and lipid profiles align with clinical trials showing ACV reduces fasting glucose and total cholesterol in diabetic patients [25,26]. Despite these insights, several limitations remain. Notably, our study did not quantify fecal SCFAs or perform untargeted profiling of hepatic bile acid metabolism—factors that may provide critical mechanistic insights into the effects of ACVP. Furthermore, the emerging evidence implicating the gut virome and mycobiome in liver pathophysiology [36] underscores the need to investigate their potential interplay with ACVP-mediated outcomes. Future mechanistic studies should employ multi-omics approaches, including fecal microbiota transplantation (FMT) experiments in germ-free murine models, to establish causal relationships between ACVP administration, microbiota composition, and metabolic improvements. Such efforts should integrate functional metagenomic analyses with in vivo validation to bridge the gaps between predictive models and biological reality. Ultimately, rigorous preclinical optimization must precede clinical translation, with clinical trials designed to evaluate ACVP’s safety profile, tolerability, and long-term efficacy in mitigating HFD-associated chronic liver pathology through gut microbiome-targeted interventions.

## Figures and Tables

**Table 1:** The composition of diets used in this study.

Ingredient (g)	AIN-93G Diet	60% High-Fat Diet
Casein, 30Mesh	200	200
L-Cystine	3	3
Corn Starch	397	0
Maltodextrin	132	125
Sucrose	100	72.8
Cellulose	50	50
Soybean Oil	70	25
t-Butylhydroquinone	0.014	0
Lard	0	245
Mineral Mix S10022M	35	0
Mineral Mix S10026B	0	50
Vitamin Mix V10037	10	0
Vitamin Mix V10001C	0	1
Choline Bitartrate	2.5	2
FD&C Blue Dye #1	0	0.05
Total weight (g)	1000	773.85
Total energy (kcal)	3850	4037.2

## Data Availability

The 16S rRNA sequencing data were uploaded to the NCBI database (SRP: PRJNA1194348). The other data will be made available on request.

## Supplementary Materials

The following supporting information can be downloaded at: https://www.mdpi.com/article/10.3390/nu17132157/s1, Figure S1: Post-hoc power analysis in metabolomics (false discovery rate (FDR) < 0.1).; Table S1: Primers for quantitative real-time PCR; Table S2: Individual weights (g) of each animal during the experiment period; Table S3: The differential metabolites among three groups.

## Abbreviations

The following abbreviations are used in this manuscript:

ALT	Alanine aminotransferase
AST	Aspartate aminotransferase
*Atg5*	*Autophagy related 5*
CHOL	Total cholesterol
CREA	Creatinine
*Dgat1*	*Diacylglycerol O-acyltransferase 1*
GLU	Glucose
HDL-C	High-density lipoprotein cholesterol
*Ho-1*	*Heme oxygenase-1*
*Hsl*	*Hormone-sensitive lipase*
*Il*	*Interleukin*
LDH	Lactate dehydrogenase
LDL-C	Low-density lipoprotein cholesterol
TGs	Triglycerides
*Tgf-β*	*Transforming growth factor-β*
UA	Uric acid
UREA	Urea
