# Supplementary material for: Apple Cider Vinegar Powder Mitigates Liver Injury in High-Fat-Diet Mice via Gut Microbiota and Metabolome Remodeling
Source: Nutrients. 2025 Jun 28;17(13):2157. doi: 10.3390/nu17132157 (PMC12251902; doi:10.3390/nu17132157)
Supplement: Supplementary file 1 [file nutrients-17-02157-s001.zip › supplementary material.pdf]

Supporting Information

Supplemental Table

Table S1 Primers for quantitative real-time PCR

Gene	Sequence of forward primers (5' to 3')	Sequence of reverse primers (5' to 3')
β-actin	CATCCGTAAAGACCTCTATGCCAAC	ATGGAGCCACCGATCCACA
Dgat1	ACCGCGAGTTCTACAGAGATTGGT	ACAGCTGCATTGCCATAGTTCCCT
Hsl	GCGCTGGAGGAGTGTTTTT	CCGCTCTCCAGTTGAACC
Atg5	AGATGGACAGCTGCACACAC	GCTGGGGGACAATGCTAATA
Il-17	CGTGGCCTCGATTGTCCGCC	GGTTTCTTAGGGGTCAGCCGCG
Muc2	GCTGACGAGTGGTTGGTGAATG	GATGAGGTGGCAGACAGGAGAC
Ocln	CGGTACAGCAGCAATGGTAA	CTCCCCACCTGTCGTGTAGT
Tgf-β	GCTCGCTTTGTACAACAGCACC	GCGGTCCACCATTAGCACG
Ho-1	GCCGAGAATGCTGAGTTCATG	TGGTACAAGGAAGCCATCACC

Table S2 Individual weights (g) of each animal during the experiment period

Group	Number	Week 0	Week 1	Week 2	Week 3	Week 4	Week 5	Week 6
CTL	1	28.76	29.68	29.89	30.24	29.14	29.09	29.59
	2	29.88	30.65	31.27	29.60	30.58	30.47	30.25
	3	25.70	26.05	26.12	27.84	26.64	26.29	26.28
	4	26.25	26.49	26.24	27.01	25.95	26.49	27.04
	5	27.92	28.67	27.81	28.81	28.93	28.37	29.38
	6	27.13	27.36	27.34	28.60	27.43	27.38	27.10
HFD	1	29.83	30.58	30.17	33.38	35.21	36.42	37.14
	2	25.29	26.63	26.42	30.71	33.78	35.84	37.06
	3	26.13	28.37	27.51	31.29	33.47	35.81	36.52
	4	28.13	29.68	29.6	33.24	35.36	38.01	38.51
	5	28.57	30.53	30.17	34.15	36.41	38.12	41.07
	6	27.7	28.37	28.78	30.98	32.09	33.86	35.5
ACVP	1	26.81	25.65	25.17	27.87	30.02	30.86	32.11
	2	27.85	28.14	27.49	28.03	30.48	31.69	32.41
	3	28.00	28.45	28.25	29.81	32.52	33.54	35.04
	4	26.52	27.00	26.38	28.70	30.13	31.05	32.57
	5	29.14	29.40	29.08	29.77	31.46	32.03	32.31
	6	27.58	28.12	27.95	29.62	30.89	30.54	31.04

Group	Number	Week 7	Week 8	Week 9	Week 10	Week 11	Week 12
CTL	1	29.88	30.41	29.87	30.42	30.68	29.82

	2	30.14	32.28	31.51	31.42	31.83	30.61
	3	26.42	27.73	27.23	27.03	27.30	26.82
	4	27.19	27.93	27.24	26.93	27.15	26.62
	5	29.33	29.85	28.97	29.34	29.56	28.50
	6	26.91	28.10	28.25	28.42	28.42	27.54
HFD	1	39.55	40.14	43.06	44.54	47.04	48.41
	2	39.7	41.27	44.12	45.76	47.28	47.58
	3	39.08	40.39	43.21	45.13	47.02	47.34
	4	40.62	42.16	44.93	45.82	46.89	47.46
	5	43.55	45.62	48.65	49.85	52.10	52.21
	6	37.26	39.11	40.73	42.21	44.76	45.06
ACVP	1	32.52	33.05	34.31	34.81	35.62	34.96
	2	33.15	34.21	34.97	35.29	36.23	35.73
	3	34.66	35.28	37.13	37.84	39.18	38.63
	4	32.69	33.14	34.34	36.43	35.67	35.41
	5	33.61	33.98	33.97	33.82	35.06	34.4
	6	32.18	32.36	33.34	33.88	34.42	33.9

Supplemental Figure

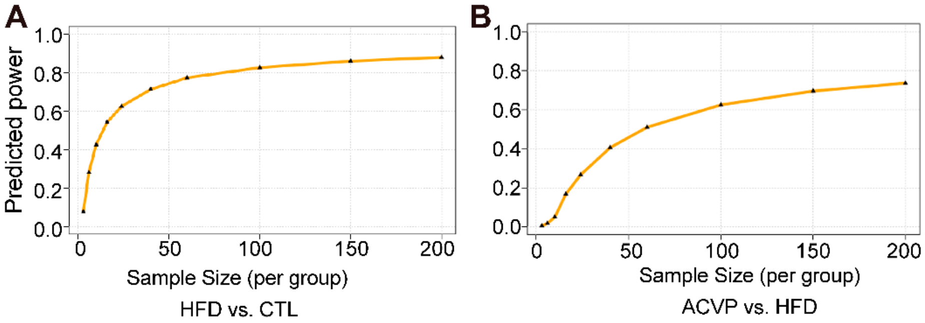

Figure S1 Post-hoc power analysis in metabolomics (false discovery rate (FDR) < 0.1).
